# Highly sensitive transient absorption imaging of graphene and graphene oxide in living cells and circulating blood

**DOI:** 10.1038/srep12394

**Published:** 2015-07-23

**Authors:** Junjie Li, Weixia Zhang, Ting-Fung Chung, Mikhail N. Slipchenko, Yong P. Chen, Ji-Xin Cheng, Chen Yang

**Affiliations:** 1 Purdue University Interdisciplinary Life Sciences Program (PULSe), Purdue University, West Lafayette, Indiana, 47907, USA; 2 Department of Chemistry, Purdue University, West Lafayette, Indiana, 47907, USA; 3 Department of Physics and Astronomy, Purdue University, West Lafayette, Indiana, 47907, USA; 4 Weldon School of Biomedical Engineering, Purdue University, West Lafayette, Indiana, 47907, USA; 5 Birck Nanotechnology Center, Purdue University, West Lafayette, Indiana, 47907, USA

## Abstract

We report a transient absorption (TA) imaging method for fast visualization and quantitative layer analysis of graphene and GO. Forward and backward imaging of graphene on various substrates under ambient condition was imaged with a speed of 2 μs per pixel. The TA intensity linearly increased with the layer number of graphene. Real-time TA imaging of GO *in vitro* with capability of quantitative analysis of intracellular concentration and e*x vivo* in circulating blood were demonstrated. These results suggest that TA microscopy is a valid tool for the study of graphene based materials.

Graphene and graphene oxide (GO) have attracted great interest and been considered as promising materials for, for example, future electronics^1^,^2^, energy or hydrogen storage^3^,^4^, biosensing^5^,^6^, and drug delivery^7^,^8^. As more applications have been explored, there is an urgent need for methods that can quantitatively and rapidly analyze graphene and graphene oxide. For graphene, its excellent electronic, optical and mechanical properties strongly depend on the number of atomic layers, thus to fabricate large scale devices on chip, it is significant to perform rapid quantitative characterization of large area graphene with layer sensitivity under ambient environment. Meanwhile, the vast applications of GO in biological environment require both visualization and quantitative analysis of its concentration, an important parameter for its biological effect^9^.

To image graphene with layer sensitivity, various microscopic and spectroscopic techniques have been developed. Atomic force microscopy^2^,^10^ and Raman spectroscopy^11^,^12^ have been reported to be reliable methods to identify and quantitatively measure the layer number of graphene. However, the low throughput preclude their use for large area scanning^10^,^13^,^14^. For example, due to the low efficiency of Raman scattering, it would take several hours to get a Raman image of an area of tens of micrometers^15^,^16^. Several electron microscopies, including low-energy electron microscopy^17^,^18^, high resolution transmission electron microscopy^2^, scanning electron microscopy^2^, scanning tunneling microscopy^19^, photoemission electron microscopy^17^, have been used for determining the number of layers of graphene. In most cases, these techniques require cumbersome sample preparation and/or high vacuum condition for characterization of limited sample area^20^. Although it is widely used for quick examination, optical microscopy can only be applied to samples deposited on properly designed substrates to get better contrast^14^,^21^. Fluorescence quenching microscopy was recently employed to quickly visualize graphene based sheets, where a fluorescent dye was coated on the surface^22^. A nonlinear optical microscopy method based on self-phase modulation was used for *in vitro* imaging of graphene, but this contrast is not sensitive to GO^23^.

GO with abundant oxygen-containing groups can be readily modified with targeting ligands to facilitate drug delivery^7^,^8^. Recently, the strong near infrared absorption of GO is utilized for the photothermal treatment of cancer or Alzheimer’s disease^24^,^25^,^26^. In spite of progresses in this biomedical direction, few methods exist for tracing GO in biological environment. Ideally, the intrinsic photoluminescence of GO can be used for cellular imaging^7^,^27^, however, the emission efficiency is low^28^,^29^. Strategies are developed to overcome such low efficiency, including fluorescently or radioactively labeling GO^25^,^30^,^31^. Radioisotopes used for radioactive labeling are hazardous and must be handled with extreme care, while fluorescence probes often introduce toxicity and interference with normal biological processes and might suffer from photobleaching^32^,^33^. What is more important, these existing methods is difficult to directly quantify the concentration of GO.

Here, we report a label-free highly sensitive imaging method for fast visualization and quantitative layer analysis of graphene and graphene oxide based on the transient absorption (TA) process. TA imaging has been developed for visualizing single nanomaterials, such as gold nanoparticles^34^,^35^, nanowires^36^, semiconductor and meta nanostructures^37^,^38^, single-walled carbon nanotubes^39^,^40^,^41^. Recently, TA spectroscopy and imaging have been employed to study the carrier dynamics in graphene^42^,^43^ and graphene oxide^44^,^45^ with limited sensitivity.

In this work, we demonstrate TA imaging with single layer sensitivity. We used megahertz modulation that effectively avoids the low-frequency laser noise and employed a resonant circuit that electronically amplified the heterodyne-detected signal. On this imaging platform, we achieved high speed (2 μs/pixel) imaging of graphene on various substrates (e.g., glass, silicon) under ambient condition and of graphene oxide in living cells and animals. The intensity of TA images is found to linearly increase with the layer number of graphene. It takes a few seconds to acquire a TA image of graphene samples, which is much faster than Raman mapping. More importantly, our method is able to image graphene and GO in biological environment with capability of quantitative analysis of intracellular concentration of well-dispersed GO functionalized with polyethylene glycol (PEG).

## Results and Discussion

**Figure 1:**
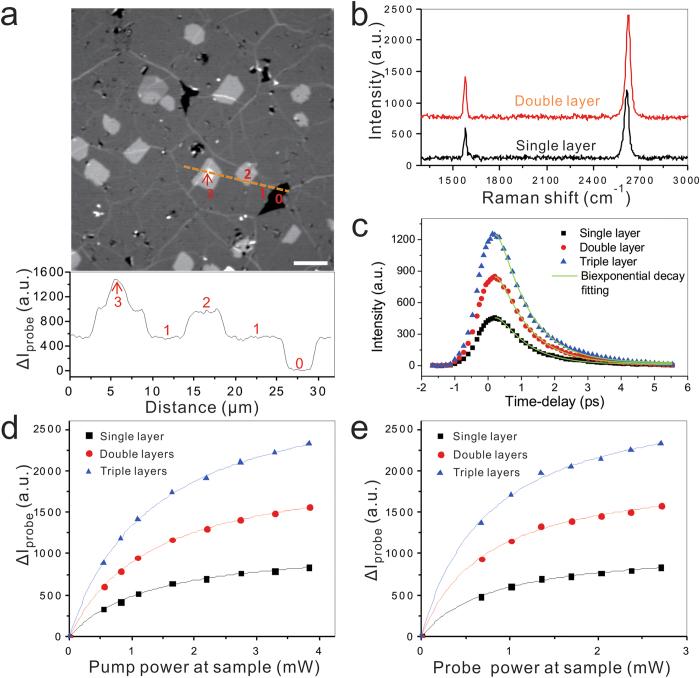
TA imaging of graphene on glass coverslip. (**a**) TA image of graphene layer (upper) and intensity profile along the dashed line (lower). Single layer (position 1) graphene can be easily distinguished from defects (position 0), double layers (position 2) and multiple layers (position 3). The pump beam was at 665 nm, and probe was at 820 nm. Scale bar: 10 μm. (**b**) Raman spectra of graphene sheet with different layer number. (**c**) Time-delay curve of TA imaging on single, double and triple graphene layers. Time zero was determined by stimulated Raman scattering (SRS) image. The data points were fitted with a two component exponential model (Green line) (**d**,**e**) Dependence of TA imaging on pump (**d**) and probe (**e**) power. Pump and Probe beam power was fixed at 1.10 mW and 0.68 mW at sample, respectively, when the other beam is tuned from 0 to 5 mW. Data were fitted with the saturation function *y* = *Ax*/(1+*x*/*x*_*s*_). Here, A is a constant, *x* is the input power, and *x*_*s*_ represents the saturation power.

TA images were acquired on a laser-scanning microscope (Fig. S1) with a pump beam and a probe beam (See Methods). Samples were prepared through transferring chemical vapor deposition grown graphene to a glass coverslip following the standard procedure^46^(See Supplementary Information). The image shown in Fig. 1a clearly revealed graphene domains with single layer (position 1 in Fig. 1a), defects (position 0), double layers (position 2) and multiple layers (position 3). The intensity profile (Fig. 1a) shows that the signal intensity *I* is quantized and linearly proportional to the number of graphene layers, *n*, with *I *= *nI*_*s*_ where *I*_*s*_ is the signal intensity from single layer graphene area. To further confirm the layer resolved by the image, we measured Raman spectra from different domains using the Raman capability on our transient absorption microscopy platform^47^. The peak ratio of G band (~1580 cm^−1^) to 2D band (~2620 cm^−1^), together with the intensity of G band and the peak shape of 2D band, was taken in consideration to confirm the layer number. The Raman spectrum (Fig. 1b) taken from the single layer domain (position 1 in Fig. 1a) exhibited a 2D/G peak ratio of 2.2 and a symmetric 2D band with full width half maximum (FWHM) of 30 cm^−1^. 2D/G peak ratio measured from 10 Raman spectra from the single layer areas was found to be 2.3 ± 0.3, which confirms the single layer assignment^11^,^48^. In addition, the intensity of G band is almost proportional to the number of graphene layers^49^, which confirmed the existence of double layer domain (position 2 in Fig. 1a). The strong, symmetric 2D band with a FWHM of 34 cm^−1^ observed from the double layers (Fig. 1b) suggested that the double layer might be rotationally mis-oriented stacked or twisted, which were found common in graphene synthesized by chemical vapor deposition^46^,^50^. We also observed broad and asymmetric 2D band from some other double layer domains (Fig. S2), suggesting Bernal stacked double layers. Collectively, the Raman spectra measurement validated the capability of TA imaging in quantitatively determining the layer number of graphene sheet. Moreover, the speed of TA imaging reported here is three orders of magnitude faster than Raman spectroscopy, which allows high-throughput mapping of large area graphene sheet in synthesis process.

A time-resolved measurement was carried out by varying time delay between pump and probe pulses. The time-resolved intensity curves obtained from single, double and triple layers (Fig. 1c) exhibited a bi-exponential decay. The fast decay (~0.2 ps) was attributed to be from coupling between excited carrier and optical phonons, while the slow decay (~1.0 ps) was considered to be from hot phonon effect^42^. These results also demonstrate the ability of TA microscopy to study the ultrafast carrier dynamics of different layer graphene in a high-throughput manner. In addition, TA imaging is not destructive to the samples, as no sample damages were observed even at the saturation power, which was 1.3 mW for pump and 0.9 mW for probe on the sample (Fig. 1d,e).

We further investigated the possible contrast mechanism of the TA signal from graphene. In a TA microscope, we measure the difference in transmission of probe (ΔI_probe_/I_probe_) induced by the presence of pump. This signal can originate from different optical processes, including optical bleach, stimulated emission, excited state absorption, and multiphoton absorption^51^. The TA signal can be positive (ΔI_probe_/I_probe_ > 0) or negative (ΔI_probe_/I_probe_ < 0) depending on different processes. For example, signals from optical bleach and stimulated emission are positive, while signal from absorption is negative. Here, we observed a positive TA signal from graphene, indicating an increase of transmitting probe induced by pump. We also measured the dependence of TA signal level on pump and probe laser intensities (Fig. 1d,e). We observed a linear relationship at low power, and saturation when pump and probe power exceeded 1.3 mW and 0.9 mW, respectively, implying an optical bleach effect was reached at high power. Thus, the major contribution to observed TA signal in graphene is likely from optical bleach due to state filling effect^37^. Our explanation is consistent with a previous study using TA imaging on semiconductor and metal nanostructures^25^.

**Figure 2:**
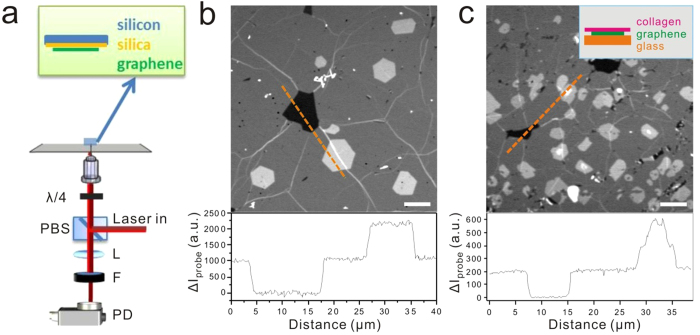
TA imaging of graphene on silicon-based sheet and with collagen coating. (**a**) Layout of epi-detection of TA signals. PBS: cube polarization beam splitter installed in interchangeable turret; λ/4: quarter-wave plate; L: lens with 100 mm focal length; F: bandpass filter used before photodiode; PD: large area photodiode. The graphene sheet was prepared on top of a thin silica layer (~300 nm), with silicon sheet underneath. (**b**) Epi-detected TA imaging of graphene and intensity profile along the dashed line. Single layer, double layers and defects were clearly resolved in a quantitative manner. The average power used for pump beam was 5 mW at 665 nm, and 10 mW for probe beam at 820 nm. Scale bar: 10 μm. (**c**) TA imaging of graphene with collagen coating and intensity profile along the dashed line. Inset: diagram of the sample preparation. Graphene sheet was placed on a glass coverslip with 0.17 mm thickness, and collagen was coated on top of the graphene sheet. Scale bar: 10 μm.

Compared to transparent substrates, non-transparent substrates, for example, SiO_2_/Si substrate, are more often required for graphene electronic applications. To demonstrate the capability of visualizing graphene sheet on SiO_2_/Si substrates, we performed TA imaging in the backward direction (Fig. 2a). In this detection scheme, photons from the specimen are collected by a large area photodiode in the backward direction, while in conventional forward detection scheme photons transmitting the specimen are collected (Fig. S1). The sample was prepared by placing the graphene sheets on a silicon substrate with a top layer of 300 nm silicon oxide (Fig. 2a). Graphene domains with different thickness, for example single layer, double layers and defects, can be readily distinguished in the image and the layer number could be quantitatively determined by the intensity profile (Fig. 2b). The results indicate TA imaging is applicable to graphene on SiO_2_/Si substrate, which would further broaden the application of this method for graphene research. In addition, we considered the rapidly increasing applications of graphene in biological environments. Recently it has been reported that using graphene as substrate enhances the differentiation of human mesenchymal stem cells into neurons^52^ and bone cells^53^. Here, we demonstrate high-quality images of graphene with collagen coating on top, mimicking the extracellular environment, could be acquired by TA microscopy (Fig. 2c). The TA signal intensities were found to be the same as those from bare graphene layers in air.

**Figure 3:**
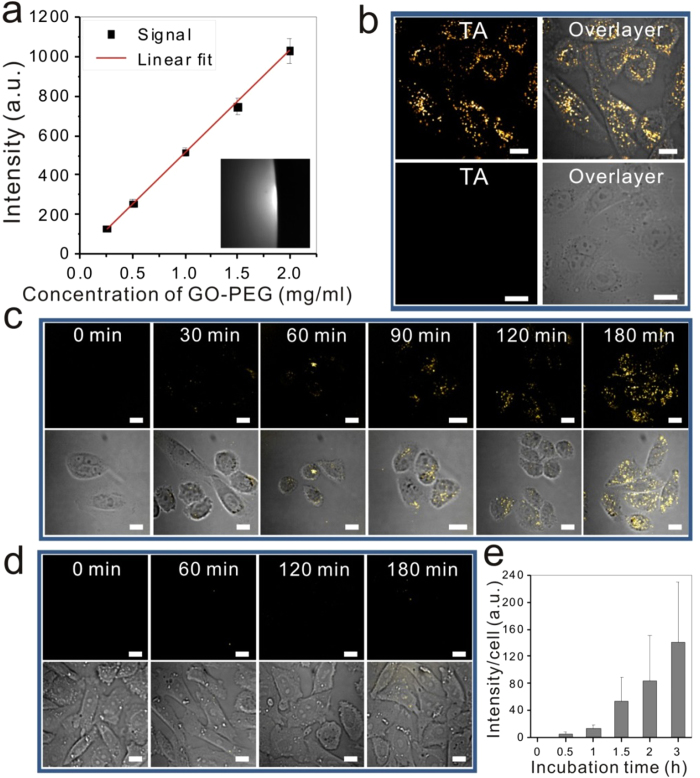
TA imaging of GO-PEG in solutions and in living cells. (**a**) TA imaging of 2 mg/ml GO-PEG aqueous solution (inset) and linear dependence of TA signal on GO-PEG solution concentration. (**b**) TA imaging of GO-PEG in living CHO cells. CHO cells were treated with 10 μg/ml GO-PEG (upper) or PEG (lower) for 3 h. Overlay of TA image (yellow dots) and transmission image (gray) showed intracellular GO-PEG in GO-PEG treated cells, but not in PEG only treated cells. Dynamic cellular uptake of GO-PEG (**c**) or PEG (**d**) was monitored by TA imaging overtime, from 0 to 180 min after treatment. TA images (first row) and overlay images (second row) showed intracellular accumulation of GO-PEG. (**e**) Quantification of mean TA signal intensity per cell in GO-PEG treated cells. Scale bar: 10 μm.

To further explore the potential of TA imaging in graphene research, we applied TA microscopy to visualize GO in living cells. GO was firstly functionalized by adding PEG tags to improve the stability and solubility of GO in aqueous solution as well as the blood circulation of GO (See Supplementary Information). After modification, GO-PEG was dissolved in aqueous solution at 2 mg/ml concentration without detectable aggregates. TA imaging of 2 mg/ml GO-PEG solution showed a uniformly distribution pattern (Fig. 3a insert). On contrary to that in graphene, the TA signal from GO-PEG solution is negative, suggesting an enhanced absorption of the probe beam happen, leading to a decrease of probe transmitting through sample. It has been shown that the band gap of different GO structures lies below 1.0 eV^54^,^55^,^56^. Our pump beam (1090 nm) energy (~1.14 eV) is enough to overcome this energy gap, and we suggest that TA signal originates from transition from localized states at the bottom of conduction band to the higher excited states.

The intensity of TA imaging was linearly proportional to the concentration of GO-PEG solution (Fig. 3a), which can be used for a quantitative analysis of intracellular GO-PEG concentration. After incubating CHO cells with 10 μg/ml GO-PEG for 3 h, TA imaging revealed an intracellular accumulation of GO-PEG in GO-PEG treated cells, but not in PEG only treated cells (Fig. 3b). The dynamic intracellular trafficking of GO was also monitored by TA imaging (Fig. S3 and the video in the Supplementary Information). To study the uptake process of GO-PEG into living cells, time-lapse imaging was performed. GO-PEG was encapsulated into the cells and accumulated in the cells over time, from the membrane to cytoplasm (Fig. 3c). On the contrary, no signal was detected from cells treated with PEG only (Fig. 3d). A quantitative analysis of the intracellular mean intensity showed an exponential increase of GO-PEG accumulation in the cells (Fig. 3e). These results indicate the TA imaging permits real-time live cell analysis of GO-PEG, which may help extend the biomedical applications of GO, including drug delivery and thermal therapy.

**Figure 4:**
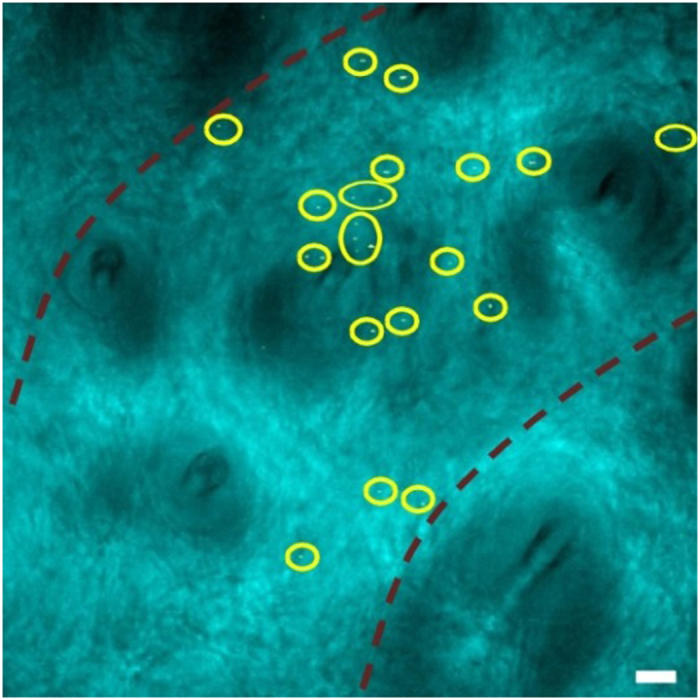
TA imaging of GO-PEG circulating in rat blood vessel. Rat was intravenously injected of 500 μl 2 mg/ml GO-PEG solution. TA imaging was performed at 30 min post injection. GO-PEG particles (yellow dots in circles) were observed in the peripheral blood vessel. Red dashed lines mark the blood vessel wall determined by transmission image. Scale bar: 20 μm.

Furthermore, we demonstrated that TA imaging could be used for *ex vivo* imaging of GO. With PEG modification, the blood circulation of GO-PEG will be enhanced, as shown in other nanosystems^57^,^58^. 30 min after intravenous injection of GO-PEG solution, the rat ear was placed under microscope for imaging. The blood vessel and surrounding tissues were visualized by transmission illumination and the circulated GO-PEG was monitored by TA imaging simultaneously (Fig. 4). As shown in Fig. 4, circulating GO-PEG particles (yellow dots in circles) in blood vessel were clearly visualized by TA imaging. Although the size of GO sheets was estimated to about 100 nm, under diffraction limit of optical microscope, the strong TA signal of GO enabled us to track even individual GO sheet, which has been demonstrated in other nanomaterials^37^,^39^,^40^. It is known that nano-GOs tend to aggregate in salt or biological solutions^7^. Thus, it is highly possible that some dots shown in Fig. 4 are aggregates but not individual GO sheet, therefore these dots varied in size and shape. Further systematic investigation will be carried out to study the related biological effects, such as bio-distribution of GO-PEG in organs. Collectively, these results provide solid evidences that TA imaging can be used to visualize and monitor the dynamics of graphene or GO in the biological environment.

## Conclusions

TA microscopy is a high-throughput versatile tool for quantitative evaluation of graphene and GO. The imaging speed is as fast as 2 μs/pixel, and it can be applied to study layer number of graphene on various substrates as well as with collagen coating. It can also be used to track GO dynamics in living cells and live animals. The high sensitivity of TA imaging method enables to detect the GO with concentration as low as 10 μg/ml, which is typical or even lower concentration used in biological experiment^9^,^24^. With the advantage of high speed, nondestructive, label-free, as well as capability of *in vitro* and *ex vivo* imaging, our study opens new opportunities for using TA imaging to facilitate the study of graphene and GO in synthesis, device fabrication, and biological applications.

## Methods

### Transient absorption microscope

The schematic of our TA microscope is shown in Fig. S1. A Ti:Sapphire laser (Spectra-Physics Lasers Inc., Mountain View, CA) pumped an optical parametric oscillator (OPO, Inspire, Spectra-Physics Lasers Inc.), producing the pump beam tunable from 490 to 750 nm and probe beam at 820 nm. Two beams were temporally synchronized and collinearly combined into a laser-scanning inverted microscope (FV300 + IX71, Olympus Inc., Central Valley, PA). The laser was focused on the sample with a 60 × water-immersed objective (NA = 1.2, UPlanApo/IR, Olympus Inc). The pump beam intensity was modulated by an acousto-optic modulator (AOM, Gooch & Housego) with about 70% modulation depth at 6 MHz or 7.08 MHz. The power of pump and probe beams was adjusted using neutral density fillers. The TA signal was detected by a photodiode (818-BB-40, Newport, CA) after a bandpass filter of 850/90 nm to block the pump beam. The photodiode output was sent to a resonant circuit (RC) which selectively transmits the heterodyne-detected TA signal at the laser modulation frequency to a fast lock-in amplifier (LIA, HF2LI, Zurich Instrument). For backward TA signal detection, a polarization beamsplitter cube (PBS) was installed in the turret to direct the signal to the photodiode. All images were acquired with 2 μs/pixel dwell time. Confocal Raman signal was detected by a spectrometer (Shamrock SR-303i-A, Andor Technology, Belfast, U.K.) mounted to the side port of the microscope. For Raman spectral analysis, a 5-ps, 80 MHz Ti:sapphire laser oscillator (Tsunami, Spectra-Physics Lasers Inc.) was used as an excitation beam at 707 nm. After acquiring the images, the turret was switched to a short-pass dichroic mirror (720dcxr, Chroma), which sent the Raman signal to the spectrometer.

### *In vitro* TA imaging of GO-PEG

Chinese hamster ovary (CHO) cells were used for imaging. CHO cells were cultured in DMEM/F-12 medium supplemented with 10% FBS at 37 °C incubator with 5% CO_2_ and were passaged every three days with a 1:5 split ratio. For imaging purpose, cells were plated into 35 mm glass-bottom dishes (*In Vitro* Scientific, D35-10-1.5-N) at density of 1 × 10^5^ cells/plate. GO-PEG solution was added to the cell culture medium 24 h after plating at a final concentration of 10 μg/ml. Cells were incubated at 37 °C for 0~3 h. Then the dishes were placed under optical microscope for imaging. For *in vitro* TA imaging, a femto-second coherent laser was used. The pump beam was at 1090 nm with average power of 20 mW at sample, and the probe beam was at 850 nm with average power of 5 mW at sample. At this frequency, the SRS signal from C-H or other bands in cells can be avoided. The scanning speed for all experiments is 2 μs/pixel.

### *Ex vivo* TA imaging of GO-PEG

All protocols were approved by the Purdue University Animal Care and Use Committee and were performed in accordance with guidelines. Adult Long-Evans rats (~300 g) were anesthetized by isoflurane. 500 μl of 2 mg/ml GO-PEG solution in PBS was injected through the jugular vein. 30 min after the injection, a small piece of rat ear was cut off and placed under the microscope for TA imaging. The imaging parameters were the same as those used in *in vitro* imaging process.

## Additional Information

**How to cite this article**: Li, J. *et al.* Highly sensitive transient absorption imaging of graphene and graphene oxide in living cells and circulating blood. *Sci. Rep.* **5**, 12394; doi: 10.1038/srep12394 (2015).

## Supplementary Material

Supplementary Information
