# Supplementary material for: Highly sensitive transient absorption imaging of graphene and graphene oxide in living cells and circulating blood
Source: Sci Rep. 2015 Jul 23;5:12394. doi: 10.1038/srep12394 (PMC5378876; doi:10.1038/srep12394)
Supplement: Supplementary Information [file srep12394-s1.pdf]

Supporting Information

Highly sensitive transient absorption imaging of graphene and graphene oxide in living cells and circulating blood

Junjie Li[1†], Weixia Zhang[2†‡], Ting-Fung Chung[3], Mikhail N. Slipchenko[4], Yong P. Chen[3,5], Ji-Xin Cheng[4*], Chen Yang[2,3] *

[1]Purdue University Interdisciplinary Life Sciences Program (PULSe), Purdue University, West Lafayette, Indiana, 47907, United States

[2]Department of Chemistry, Purdue University, West Lafayette, Indiana, 47907, United States

[3]Department of Physics, Purdue University, West Lafayette, Indiana, 47907, United States

[4]Weldon School of Biomedical Engineering, Purdue University, West Lafayette, Indiana, 47907, United States

[5]Birck Nanotechnology Center, Purdue University, West Lafayette, Indiana, 47907, United States

[†]These authors contributed equally to the study.

[‡]Currently address: School of Engineering and Applied Sciences, Harvard University, Cambridge, Massachusetts, 02138, United States

*Corresponding author: yang@purdue.edu, jcheng@purdue.edu

Materials and methods

Graphene synthesis[1]. Graphene samples were grown on 25 μm thick of Cu foils (Sigma-Aldrich, 99.98%) by CVD at ambient pressure. The Cu foil was loaded into a CVD furnace and heated to 1000 °C in 50 sccm of flowing H_2. After 1000 °C was attainted, the foil was annealed for 1 hour. Then 10 sccm CH_4 was flowed for 30 min. The system was then cooled to room temperature.

Graphene transfer[2]. The PMMA-mediated transfer procedure starts with coating one side of the as-synthesized graphene with PMMA(Microchem) resist. After being cured at 180 °C for 1 min, the another side of the sample was treated with O_2 plasma to remove the graphene on that side. The sample was then left in Ammonium persulfate (VWR, 98%) solution for overnight to completely dissolve away the copper layer. Then the graphene was transferred to another substrate (e.g. glass coverslip). The PMMA coating was removed with acetone and isopropanol.

Synthesis of graphite oxide (GO)[3, 4]. GO was prepared by a modified Hummers method using graphite powders (Bay carbon, SP-1). In a typical reaction, 23 ml H_2SO_4 was added to a mixture

of graphite flakes (0.5 g) and $NaNO_3$ (0.5 g), and the mixture was placed in an ice bath. Then 3 g of KMnO4 was slowly added. Once mixed, the solution was transferred to a 35 ^oC water bath and stirred for about 1 h, at which time 40 ml of water was added slowly and the solution was stirred for 30 min while the temperature was raised to 90 ^oC. Finally, additional 100 ml of water and 3 ml of H2O2 (30%) were added. The warm solution was then filtered and washed with 100 ml of water. The filter cake was then resuspended in water by mechanical agitation. Low-speed centrifugation (1000 rpm) was used and repeated (3-5 times) to remove all visible particles from the precipitates. The supernatant then underwent two more high-speed centrifugation (8000 rpm) steps for 15 min to remove small GO pieces and water-soluble byproduct. The final sediment was redispersed in water with sonication using an ultrasonic cleaner, giving a solution of exfoliated GO (~ 100 nm) with concentration of 2 mg/ml.

Pegylation of graphene oxide[5, 6]. For pegylation, GO suspension (5 ml) was bath sonicated for 1 h to give a clear solution. 1.2 g of NaOH and 1.0 g of chloroacetic acid were added to the GO suspension and bath sonicated for 3 h to convert the –OH groups to –COOH via conjugation of acetic acid moieties giving GO-COOH. The resulting GO-COOH solution was neutralized using hydrochloric acid and purified by repeated rinsing and centrifugation. The GO-COOH suspension was diluted by a factor of 2, and 24 mg of 6-arm polyethylene glycol-amino (Sunbio Inc.) was added to 6 ml of diluted GO suspension. The mixture was sonicated for 10 min. Then N-(3-dimethylaminopropyl-N'-ethylcarbodiimide) hydrochloride (EDC, from Sigma Inc.) was added in two portions to give a concentration of 4 mmol/L, the mixture was allowed to react overnight. The final product (GO-PEG) was obtained by ultracentrifugation at 45000 rpm in phosphate buffered saline (PBS) solution for 1 h, saving the supernatant (yield 50%), which was stored at 4 ^oC. The aggregates were discarded.

1. Li, J., Chung, T.-F., Chen, Y.P. & Cheng, G.J. Nanoscale Strainability of Graphene by Laser Shock-Induced Three-Dimensional Shaping. *Nano Lett.* 12, 4577-4583 (2012).
2. Lee, S., Lee, K. & Zhong, Z. Wafer Scale Homogeneous Bilayer Graphene Films by Chemical Vapor Deposition. *Nano Lett.* 10, 4702-4707 (2010).
3. Cote, L.J., Kim, F. & Huang, J. Langmuir−Blodgett Assembly of Graphite Oxide Single Layers. *J. Am. Chem. Soc.* 131, 1043-1049 (2009).
4. Hummers, W.S. & Offeman, R.E. Preparation of Graphitic Oxide. *J. Am. Chem. Soc.* 80, 1339-1339 (1958).
5. Sun, X. et al. Nano-graphene oxide for cellular imaging and drug delivery. *Nano Res* 1, 203-212 (2008).
6. Yang, K. et al. In Vivo Pharmacokinetics, Long-Term Biodistribution, and Toxicology of PEGylated Graphene in Mice. *ACS Nano* 5, 516-522 (2010).

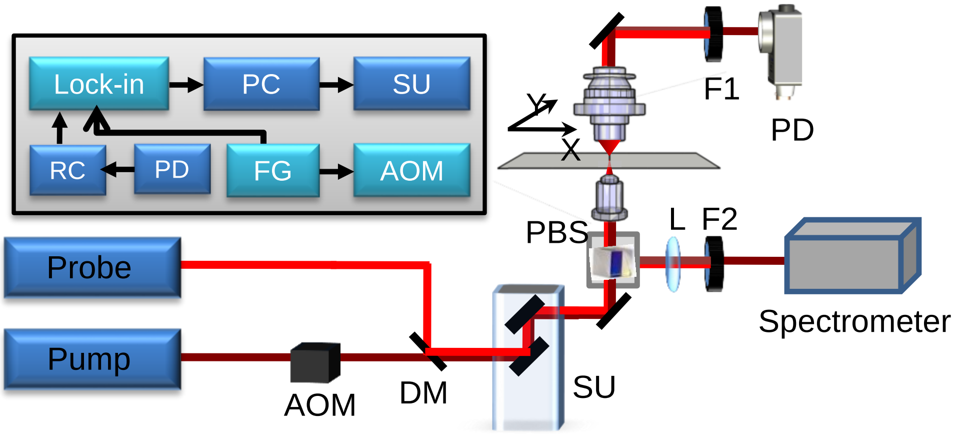

Figure S1. Layout of our TA imaging system setup. AOM: acousto-optic modulator; DM: diachroic mirror; SU: scanning unit; L: lens of 100 mm focal length; PD1 and PD2: photodiode; F1 and F2: bandpass filters; PBS: cube polarization beam splitter; RC: resonant circuit; FG: function generator.

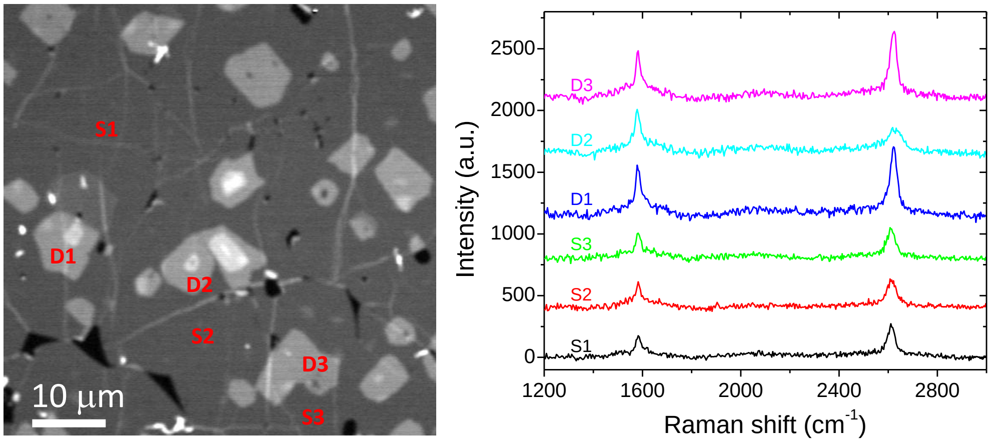

Figure S2. Multiple Raman spectra taken from graphene domains with the same layer number. Raman spectra were acquired at positions on single layer domain (marked in the image as S1-3) and double layer domain (D1-3). Spectra were offset for clarity. The 2D band of spectra taken from monolayer domain had similar profiles. However, spectra taken from bilayer domain had two kinds of different 2D bands, indicating the existence of two types of bilayers. Scale bar: 10 µm

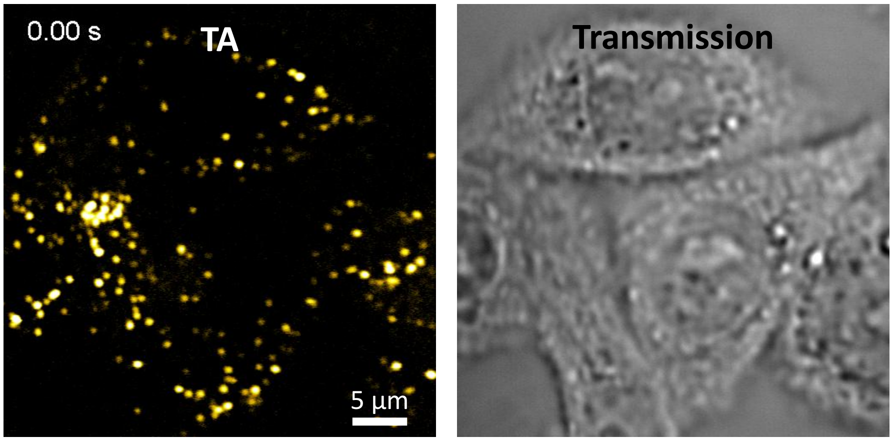

Figure S3. Real-time TA imaging of GO-PEG in living CHO cells. The video contains 50 frames with around 1 s per frame. Yellow dots indicate the GO-PEG accumulation in cells. Gray is the transmission image. Scale bar: 5 µm.
